# Prevalence and clinical correlates of hyperhomocysteinemia in Chinese urban population with hypertension

**DOI:** 10.3389/fendo.2024.1369997

**Published:** 2024-02-20

**Authors:** Yayun Xu, Haixing Feng, Liping Zhang, Yanlei Li, Feng Chi, Lijie Ren

**Affiliations:** Department of Neurology, The First Affiliated Hospital of Shenzhen University, Shenzhen Second People’s Hospital, Shenzhen, China

**Keywords:** hyperhomocysteinemia, hypertension, urban, prevalence, clinical correlates

## Abstract

**Context:**

The coexistence of hypertension and elevated homocysteine (Hcy) levels has a mutually reinforcing impact on the susceptibility to cardio-cerebrovascular disease.

**Objective:**

The aim was to assess the prevalence, clinical correlation, and demographic characteristics of hyperhomocysteinemia (HHcy) within the Chinese urban population with hypertension.

**Methods:**

A cohort of 473 individuals with hypertension were selected from four communities in Shenzhen, China. Demographic attributes, clinical profiles, and lifestyle behaviors were gathered and compared between individuals with and without HHcy. A logistic regression model was employed to examine potential factors associated with the prevalence of HHcy. Correlation between Hcy levels and clinical characteristics was assessed through multiple linear regression analysis.

**Results:**

The prevalence of HHcy in the population with hypertension was 31.3%. In comparison to individuals without HHcy, those with HHcy exhibited a higher proportion of males, a higher prevalence of smoking and alcohol consumption, and a higher proportion of cases with the homozygous (TT) genotype at the MTHFR C677T polymorphism. Moreover, individuals with HHcy had lower levels of folic acid (FA), and lower fruit and vitamin B12 intake. Furthermore, the risk factors for HHcy were male (*B* = 1.430, OR = 4.179) and MTHFR (TT) (*B* = 1.086, OR = 2.961). In addition, the multiple linear regression analysis revealed a significant association between Hcy levels and gender (*B* = -2.784, *P* = 0.004), MTHFR genotypes (*B* = 1.410, *P* = 0.005), and FA levels (*B* = -0.136, *P* = 0.030).

**Conclusion:**

The high prevalence of HHcy among hypertensive patients in this Chinese urban population underscores the necessity for interventions targeting modifiable risk factors such as dietary choices and lifestyle practices.

## Introduction

In the past three decades, there has been a persistent global rise in the prevalence of cardio-cerebrovascular disease. Specifically, cardiovascular disease (CVD) persists as the leading cause of mortality and morbidity on a global scale. Over the course of the last three decades, the burden of CVD has consistently escalated, with a notable 53.7% increase in the number of deaths (from 12.1 million in 1990 to 18.6 million in 2019) and a substantial 94.4% rise in years lived with disability (from 17.7 million in 1990 to 34.4 million in 2019) (1). Significantly, China experienced the highest number of CVD fatalities, with CVD ranking as the primary cause of death in both rural and urban regions, constituting 46.7% and 44.3% of total mortalities in 2019, respectively (2). Similarly, cerebrovascular disease ranks as the second most lethal ailment on a global scale, and acute cerebrovascular disease exhibits a higher incidence of disability compared to any other individual disease, thereby imposing substantial societal burdens (3). Hypertension has emerged as the primary risk factor for cardio-cerebrovascular disease (4). Due to the widespread adoption of unhealthy lifestyles resulting from rapid economic development and the accelerated aging process, hypertension has witnessed an increase in low- and middle-income countries, including China (5). According to recent survey data from China, the prevalence of hypertension among individuals aged 18 years from 2012 to 2015 reached a significant rate of 27.9% (6). Unfortunately, the existing measures for preventing and controlling hypertension are inadequate (7, 8). Consequently, determining the most effective approach to enhance hypertension management and alleviate the burden of cardio-cerebrovascular disease is a significant global concern.

Homocysteine (Hcy) is a sulfur-containing intermediary amino acid generated through the metabolic conversion of methionine to cysteine. The condition of having elevated plasma concentrations of Hcy above the established normal threshold of 15 μmol/l is referred to as hyperhomocysteinemia (HHcy) (9, 10). HHcy could potentially have detrimental effects on individuals with hypertension, as it collaboratively enhances the risk of cardio-cerebrovascular disease through a multiplicative effect (11, 12). At present, the specific mechanisms by which HHcy influences the susceptibility to hypertension and cardio-cerebrovascular disease remain uncertain. Several hypotheses have been proposed. HHcy has been suggested to potentially elevate the likelihood of developing hypertension and cardio-cerebrovascular disease through the subsequent mechanisms (13): (1) elevation of arterial blood pressure; (2) induction of endothelial dysfunction by augmenting oxidant stress and reducing nitric oxide release, thereby impairing vasodilation; (3) provoking oxidative damage to vascular endothelial cells and hindering the synthesis of nitric oxide, a potent vasodilator, by the endothelium; or (4) augmenting platelet adhesion to endothelial cells, thereby facilitating the proliferation of vascular smooth muscle cells. A nested case-control study conducted in China, involving a cohort of 39,165 Chinese participants who were followed for an average duration of 6.2 years, revealed that individuals diagnosed with hypertension and exhibiting elevated levels of Hcy faced a significantly heightened risk of stroke and stroke-related mortality (14). Compared to those with normal blood pressure and Hcy levels, the aforementioned group experienced an 11.7-fold increase in the likelihood of stroke and a 10.7-fold increase in the likelihood of stroke-related death (14). More recently, a retrospective cohort study conducted in China, involving a total of 1226 participants and a follow-up period of 17 years, presents compelling evidence indicating that individuals with hypertension and an Hcy level exceeding 15 μmol/L had a significantly higher risk of developing stroke and cardiovascular diseases compared to those with hypertension and an Hcy level below 10  μmol/L (15). The adjusted hazard ratios for stroke and cardiovascular diseases were found to be 2.12 and 2.24, respectively (15). Therefore, the identification of distinct risk factors associated with HHcy in hypertensive patients holds significant academic importance in mitigating the occurrence of cardio-cerebrovascular events.

In China, the prevalence of H-type hypertension, characterized by hypertension and HHcy, is observed to be higher compared to other populations (16). This can be attributed to inadequate dietary intake of folic acid (FA) and B vitamins, as well as a high mutation rate of the methylenetetrahydrofolate reductase (MTHFR) gene (the C677T single nucleotide polymorphism), leading to elevated levels of Hcy (17, 18). In the present study, we evaluated the prevalence, clinical association, and demographic attributes of HHcy among hypertensive individuals in the Chinese urban population, with the aim to identify specific risk factors of HHcy in hypertensive patients and provide intervention and management strategies for cardio-cerebrovascular disease.

## Methods

### Subjects

This study employed a cross-sectional observational design and involved the recruitment of 473 individuals diagnosed with hypertension from four communities in Shenzhen, China. Inclusion criteria for all participants encompassed: (1) being over 18 years of age; (2) belonging to the Han Chinese population; (3) adhering to the Chinese Guidelines for the Prevention and Treatment of Hypertension (2018 Revised Edition); (4) being local residents. Exclusion criteria for all participants encompassed: (1) the existence of a neurological diagnosis, a serious medical condition, or an unstable medical condition; (2) a diagnosis of major psychiatric disorders such as schizophrenia and major depressive disorder, severe somatic disease, or substance abuse. The study protocol received approval from the Clinical Research Ethics Committee of Shenzhen Second People’s Hospital (ID number: 20210824001) and was conducted in adherence to the principles outlined in the Declaration of Helsinki. Prior to participation, all participants provided written informed consent subsequent to receiving a comprehensive explanation of the study.

### Demographic and clinical data collection

Each participant completed a comprehensive questionnaire capturing general information, sociodemographic characteristics, and lifestyle behaviors. Additionally, a physical examination was conducted, encompassing assessments of height, weight, body mass index (BMI), heart rate, and blood pressure measurements. Furthermore, fasting venous blood samples were collected from each subject between 8:00 and 9:00 AM. Subsequently, the blood samples were subjected to centrifugation at a speed of 2500 revolutions per minute for a duration of 20 minutes at a temperature of 4°C. The resulting serum was then collected and preserved at a temperature of -80°C until the time of analysis. Biochemical parameters pertaining to liver and kidney function, serum lipids, and glucose levels were quantified using an automated biochemical analyzer. Plasma Hcy, folic acid (FA), vitamin B12 and vitamin D levels, and methylenetetrahydrofolate reductase (MTHFR) C677T and reduced folate carrier (RFC) G80A genotypes were determined by the Shenzhen Tailored Medical Laboratory (Shenzhen, China). In terms of MTHFR C677T genotype and RFC G80A genotype, they were analyzed using a fluorescence PCR detection kit (PCR-fluorescence probe). Specifically, Fluorescence PCR detection was performed using a 4-μl whole genome DNA sample and a 10-μl PCR reaction system. The denaturation step was carried out at 95°C for 15 s, followed by annealing/extension at 60°C for 60 s, as recommended by the manufacturer. The reaction was subjected to 45 cycles. Following the completion of the PCR, the endpoint fluorescence in each well of the samples was quantified utilizing the ABI 7500 fluorescence quantitative PCR instrument (Applied Biosystems, Foster City, CA, USA). Subsequently, the genotyping outcomes were precisely ascertained employing the ABI 3730 Genetic Analyzer (Applied Biosystems, Foster City, CA, USA).

### Statistical analysis

The data analysis was carried out utilizing SPSS (Version 17.0; SPSS, Inc, Chicago, IL, USA). The Kolmogorov-Smirnov one-sample test was utilized to identify normal distributions. Descriptive analyses were conducted, with normally distributed quantitative variables presented as means ± standard deviation (SD), and skewed quantitative variables presented as median and interquartile range (IQR; 25-75%). The Chi-square test was employed for categorical variables. To account for multiple testing, the Bonferroni correction was implemented. In order to investigate the risk factors associated with HHcy in individuals diagnosed with hypertension, the study employed univariate analyses to compare subjects with HHcy to those without HHcy. Variables with a significance level of *P* < 0.1 were further analyzed using logistic regression analyses. Additionally, Spearman correlation analysis and multiple linear regression analysis were conducted to examine the associations between Hcy levels and clinical factors. Statistical significance was determined at a *P*-value of less than 0.05 (two-tailed). Goodness-of-fit test based on the Chi-square was used to determine whether the genotype frequency distribution conformed to Hardy-Weinberg equilibrium (HWE). Sample size calculation was calculated using the G*Power 3.1 software, with power set at 0.99 to detect the medium-effect size (d = 0.50) and alpha = 0.01 with allocation ratio N2/N1 of 2. Considering a previous study involving 5935 hypertensive patients in China demonstrated a HHcy prevalence of 31.4% (13), the allocation ratio N2/N1 was set at 2. The required sample size was 436, with 145 subjects with HHcy and 291 subjects without HHcy. In the present study, a total of 473 individuals diagnosed with hypertension were enrolled, with 148 subjects with HHcy and 325 subjects without HHcy.

## Results

### Prevalence of HHcy in Chinese urban population with hypertension

HHcy was operationally defined as plasma Hcy levels exceeding 15 μmol/L. Consequently, a total of 473 hypertensive individuals were enrolled in this study, of which 148 (31.3%) fulfilled the diagnostic criteria for HHcy.

### Differences in demographic characteristics, clinical profiles, distribution of MTHFR and RFC genotypes, and lifestyle behaviors of subjects with or without HHcy

According to the data presented in **Table 1**, there is a notable disparity in the gender distribution between individuals with and without HHcy. The proportion of males is significantly higher (*χ^2^* = 68.237, *P* < 0.001, OR = 7.110; 95% confidence interval (CL): 4.30-11.76) among those with HHcy (85.1%) compared to those without HHcy (44.6%). Moreover, individuals with HHcy exhibit significantly lower levels of FA (*Z* = -3.511, *P* < 0.001) in comparison to individuals without HHcy.

**Table 1:** Demographic characteristics and clinical profiles of subjects with or without HHcy.

	Subjects without HHcy n = 325	Subjects with HHcy n = 148	*Z*	*P*
Age (years)	53 (48, 59)	54 (49, 59.75)	-1.009	0.313
BMI (kg/m^2^)	25.23 (23.38, 27.66)	25.68 (23.54, 28.02)	-0.536	0.592
Male, n (%)	145 (44.6%)	126 (85.1%)	68.237	<0.001
SBP (mmHg)	131.50 (125.00, 141.75)	133.50 (124.75, 143.25)	-0.948	0.343
DBP (mmHg)	83.00 (77.00, 89.50)	84.50 (78.25, 91.50)	-1.522	0.128
LSBP (mmHg)	131 (124, 141.5)	132 (125, 143)	-0.870	0.384
LDBP (mmHg)	83 (76, 89)	85 (78, 91)	-1.662	0.097
Heart rate (beats per minute)	75 (71, 81)	77 (71, 85)	-1.843	0.065
RSBP (mmHg)	132 (126, 142.5)	133 (127, 145.5)	-0.865	0.387
RDBP (mmHg)	83 (78, 90)	85 (78, 91)	-1.178	0.239
GLU (mmol/L)	5.22 (4.80, 6.10)	5.20 (4.80, 6.14)	-0.320	0.749
ALT (U/L)	21.5 (16.0, 35.0)	20.0 (14.0, 29.75)	-1.466	0.143
AST (U/L)	19.0 (16.0, 24.0)	19.0 (16.0, 22.0)	-1.357	0.175
ALB (g/L)	47.1 (45.6, 49.0)	47.65 (46.12, 49.1)	-1.254	0.210
TBIL (μmol/L)	11.0 (8.3, 14.3)	10.6 (8.9, 14.1)	-0.352	0.725
DBIL (μmol/L)	3.45 (2.60, 4.42)	3.3 (2.60, 4.35)	-0.449	0.653
BUN (μmol/L)	5.4 (4.4, 6.5)	5.6 (4.45, 6.9)	-0.593	0.553
CREA (μmol/L)	68 (54, 81)	69.5 (52.5, 85.25)	-0.907	0.364
TCHO (mmol/L)	4.62 (3.94, 5.43)	4.54 (3.83, 5.43)	-0.672	0.502
TG (mmol/L)	1.55 (1.04, 2.29)	1.37 (1.07, 1.49)	-0.066	0.947
HDL (mmol/L)	1.27 (1.05, 1.49)	1.21 (1.07, 1.49)	-0.257	0.797
LDL (mmol/L)	2.92 (2.16, 3.61)	2.71 (2.13, 3.50)	-0.685	0.493
FA (ng/ml)	10.43 (6.47, 15.15)	8.29 (4.52, 12.86)	-3.511	<0.001
Vitamin B12 (pg/ml)	339.4 (193.05, 433.37)	274.40 (191.07, 425.87	-0.817	0.414
Vitamin D (ng/ml)	16.61 (12.61, 21.39)	17.77 (13.74, 22.06)	-1.618	0.106
Hcy (μmol/L)	11.35 (9.41, 12.97)	18.25 (16.40, 20.63)	-17.448	<0.001

BMI, body mass index; SBP, systolic blood pressure; DBP, diastolic blood pressure; LSBP, left ventricular systolic pressure; LDBP, left diastolic blood pressure; RSBP, right systolic blood pressure; RDBP, right diastolic blood pressure; GLU, glucose; ALT, alanine transaminase; AST, aspartate aminotransferase; ALB, albumin; TBIL, total bilirubin; DBIL, direct bilirubin; BUN, Blood urea nitrogen; CREA, creatinine; TCHO, Total cholesterol; TG, triglyceride; HDL-C, high-density lipoprotein cholesterol; LDL-C, low-density lipoprotein cholesterol; FA, folic acid; Hcy, homocysteine. Units of measurement are reported in **Table 1** where applicable.

As shown in **Table 2**, the results of Goodness-of-fit test based on the Chi-square showed that the genotype frequency distribution of RFC G80A and MTHFR C677T conformed to HWE, and the difference was not statistically significant (*P* > 0.05), which demonstrated that the study objects were community representative. According to the data presented in **Table 3**, CT (OR = 1.611, 95% CI = 1.038-2.501, *P* = 0.034) and TT (OR = 3.054, 95% CI = 1.658-5.624, *P* < 0.001) genotypes of MTHFR C677T polymorphism increased the risk of HHcy compared to CC genotype. The RFC G80A variant was not associated with HHcy in any inheritance models tested (co-dominant, dominant and recessive).

**Table 2:** Hardy-Weinberg equilibrium (HWE) results of RFC G80A and MTHFR C677T variants polymorphisms.

Gene	Genotype	Actual frequencies n (%)	Predicted frequencies n	χ^2^	*P*
RFC G80A	GG	21.90 (99)	23.5	0.372	0.830
	GA	52.88 (239)	50.0		
	AA	25.22 (114)	26.6		
MTHFR C677T	CC	49.78 (225)	47.3	1.258	0.533
	CT	38.05 (172)	42.9		
	TT	12.17 (55)	9.7		

**Table 3:** Genotypic and allelic frequencies of RFC and MTHFR variants polymorphisms in subjects with or without HHcy.

Polymorphism	Subjects without HHcy n = 325	Subjects with HHcy n = 148	OR (95% CI)	*P*
RFC G80A				
Codominant				
GG	66 (21.1%)	33 (23.7%)	1.00	–
GA	170 (54.3%)	69 (49.6%)	0.812 (0.491, 1.342)	0.416
AA	77 (24.6%)	37 (26.6%)	0.961 (0.542, 1.704)	0.892
Dominant				
GG	66 (21.1%)	33 (23.7%)	1.00	–
GA+AA	247 (78.9%)	106 (76.3%)	0.858 (0.533, 1.381)	0.529
Recessive				
GG+GA	236 (75.4%)	102 (73.4%)	1.00	–
AA	77 (24.6%)	37 (26.6%)	1.112 (0.705, 1.754)	0.649
Allele				
G	302 (48.2%)	135 (48.6%)	1.00	–
A	324 (51.8%)	143 (51.4%)	0.993 (0.717, 1.374)	0.966
MTHFR C677T				
Codominant				
CC	171 (54.6%)	54 (38.8%)	1.00	–
CT	114 (36.4%)	58 (41.7%)	1.611 (1.038-2.501)	0.034
TT	28 (8.9%)	27 (19.4%)	3.054 (1.658-5.624)	< 0.001
Dominant				
CC	171 (54.6%)	54 (38.8%)	1.00	–
CT+TT	142 (45.4%)	85 (61.2%)	1.896 (1.261-2.848)	0.002
Recessive				
CC+CT	285 (91.1%)	112 (80.6%)	1.00	–
TT	28 (8.9%)	27 (19.4%)	2.454 (1.385-4.348)	0.002
Allele				
C	456 (72.8%)	166 (59.7%)	1.00	–
T	170 (27.2%)	112 (40.3%)	1.523 (1.077-2.154)	0.017

–, not given.

In terms of lifestyle behaviors, individuals with HHcy exhibited a greater prevalence of tobacco smoking (*χ^2^* = 30.103, *P* < 0.001) and alcohol use (*χ^2^* = 33.922, *P* < 0.001), as well as a lower intake of fruit (*χ^2^* = 8.141, *P* = 0.017) and vitamin B12 (*χ^2^* = 5.298, *P* = 0.021), in comparison to those without HHcy (**Table 4**).

**Table 4:** Lifestyle behaviors of subjects with or without HHcy.

	Subjects without HHcy n = 325	Subjects with HHcy n = 148	χ^2^	*P*
Exercise			3.229	0.358
no exercise	59 (18.8%)	32 (23.4%)		
occasional	41 (13.1%)	12 (8.6%)		
more than once a week	41 (13.1%)	14 (10.2%)		
daily	173 (55.1%)	79 (57.7%)		
Vegetable consumption			2.765	0.251
≤ 2 days/week	19 (6.4%)	11 (9.2%)		
3-4 days/week	15 (5.1%)	10 (8.3%)		
≥ 5 days/week	262 (88.5%)	99 (82.5%)		
Fruit consumption			8.141	0.017
≤ 2 days/week	96 (32.4%)	54 (45.4%)		
3-4 days/week	49 (16.6%)	22 (18.5%)		
≥ 5 days/week	151 (51.0%)	43 (36.1%)		
Types of staple foods			3.764	0.052
mainly rice	284 (96.3%)	110 (91.7%)		
mainly pasta	11 (3.7%)	10 (8.3%)		
Meat consumption			2.718	0.437
basically not eating	30 (10.2%)	11 (9.2%)		
1-2 times/week	25 (8.5%)	9 (7.5%)		
3-5 times/week	42 (14.3%)	25 (20.8%)		
6 times/week	197 (67.0%)	75 (62.5%)		
Type of meat			6.855	0.077
no meat	15 (5.1%)	5 (4.3%)		
mainly lean meat	212 (71.6%)	71 (60.7%)		
eat both fat and lean meat	60 (20.3%)	33 (28.2%)		
mainly fat meat	9 (3.0%)	8 (6.8%)		
Tofu consumption			1.717	0.633
basically not eating	175 (59.3%)	64 (54.2%)		
1-2 times/week	96 (32.5%)	45 (38.1%)		
3-5 times/week	19 (6.4%)	6 (5.1%)		
≥ 6 times/week	5 (1.7%)	3 (2.5%)		
Nut consumption			1.396	0.706
basically not eating	230 (78.0%)	93 (78.8%)		
1-2 times/week	37 (12.5%)	14 (11.9%)		
3-5 times/week	16 (5.4%)	4 (3.4%)		
≥ 6 times/week	12 (4.1%)	7 (5.9%)		
Beverage consumption			6.658	0.084
basically not drinking	261 (88.2%)	94 (79.7%)		
1-2 times/week	19 (6.4%)	13 (11.0%)		
3-5 times/week	8 (2.7%)	3 (2.5%)		
≥ 6 times/week	8 (2.7%)	8 (6.8%)		
Water consumption			3.294	0.349
basically not drinking	3 (1.0%)	0 (0.0%)		
1-2 times/week	4 (1.4%)	0 (0.0%)		
3-5 times/week	12 (5.4%)	3 (2.6%)		
≥ 6 times/week	276 (93.6%)	111 (97.4)		
Kitchen type			3.695	0.158
open	90 (30.6%)	33 (28.0%)		
semi-open	120 (40.8%)	40 (33.9%)		
closed	84 (28.6%)	45 (38.1%)		
Dietary habit			2.387	0.303
cooking at home	266 (90.8%)	102 (86.4%)		
takeout eating	1 (0.3%)	0 (0.0%)		
dining out	26 (8.9%)	16 (13.6%)		
Vitamin B12 supplements			5.298	0.021
no	238 (81.2%)	105 (90.5%)		
yes	55 (18.8%)	11 (9.5%)		
Smoking			30.103	<0.001
never smoke	220 (73.3%)	63 (47.0%)		
quit smoking	29 (9.7%)	19 (14.2%)		
always smoke	51 (17.0%)	52 (38.8%)		
Frequency of alcohol consumed			33.922	<0.001
never	240 (78.9%)	73 (54.9%)		
occasionally	53 (17.4%)	43 (32.3%)		
frequently	10 (3.3%)	9 (6.8%)		
every day	1 (0.3%)	8 (6.0%)		
Alcohol drinking status			27.504	<0.001
never drinks alcohol	240 (78.9%)	73 (54.9%)		
quit drinking	20 (6.6%)	14 (10.5%)		
drinking	44 (14.5%)	46 (34.6%)		
Use of antihypertensive drugs			0.277	0.599
no	32 (10.4%)	17 (12.1%)		
yes	276 (89.6%)	124 (87.9%)		

### Risk factors for HHcy in Chinese urban population with hypertension

A binary logistic regression model was applied to detect risk factors for HHcy (subjects without HHcy = 0, subjects with HHcy = 1), including gender (assignment: male = 1, female = 2), heart rate, FA, MTHFR genotypes (assignment: CC = 1, CT = 2, TT = 3), fruit consumption (assignment: ≤ 2 days/week = 1, 3-4 days/week = 2, ≥ 5 days/week = 3), types of staple foods (assignment: mainly rice = 1, mainly pasta = 2), type of meat (assignment: no meat = 1, mainly lean meat = 2, eat both fat and lean meat = 3, mainly fat meat = 4), beverage consumption (assignment: basically not drinking = 1, 1-2 times/week = 2, 3-5 times/week = 3, ≥ 6 times/week = 4), vitamin B12 supplements (assignment: no = 0, yes = 1), smoking (assignment: never smoke = 1, quit smoking = 2, always smoke = 3), frequency of alcohol consumed (assignment: never = 1, occasionally = 2, frequently = 3, every day = 4), and alcohol drinking status (assignment: never drinks alcohol = 1, quit drinking = 2, drinking = 3). As shown in **Table 5**, the risk factors for HHcy were as follows: male (*B* = 1.430, *P* < 0.001, OR = 4.179, 95%CL: 2.005-8.707) and MTHFR (TT) (*B* = 1.086, *P* = 0.006, OR = 2.961, 95%CL: 1.357-6.464).

**Table 5:** Binary logistic regression analysis of potential risk factors related to HHcy in the hypertension population.

	*B*	*S.E.*	*P*	Exp (B)	*95% CL*
Gender (male)	1.430	0.375	< 0.001	4.179	2.005-8.707
LDBP	-0.007	0.013	0.569	0.993	0.968-1.018
Heart rate	0.020	0.012	0.088	1.020	0.997-1.043
FA	-0.019	0.024	0.415	0.981	0.936-1.028
MTHFR (CC)			0.017		
MTHFR (CT)	0.520	0.295	0.078	1.682	0.943-3.000
MTHFR (TT)	1.086	0.398	0.006	2.961	1.357-6.464
Fruit consumption	-0.089	0.153	0.561	0.915	0.677-1.235
Type of staple food	0.614	0.589	0.298	1.847	0.582-5.859
Type of meat	0.149	0.218	0.494	1.161	0.757-1.779
Beverage consumption	0.222	0.182	0.222	1.249	0.874-1.785
Vitamin B12 supplements	-0.884	0.476	0.064	0.413	0.162-1.051
Smoking	0.079	0.174	0.651	1.082	0.769-1.523
Frequency of alcohol consumed	0.684	0.352	0.052	1.981	0.993-3.951
Alcohol drinking status	-0.271	0.292	0.353	0.762	0.430-1.352

### Factors influencing Hcy levels in Chinese urban population with hypertension

**Figure 1:**
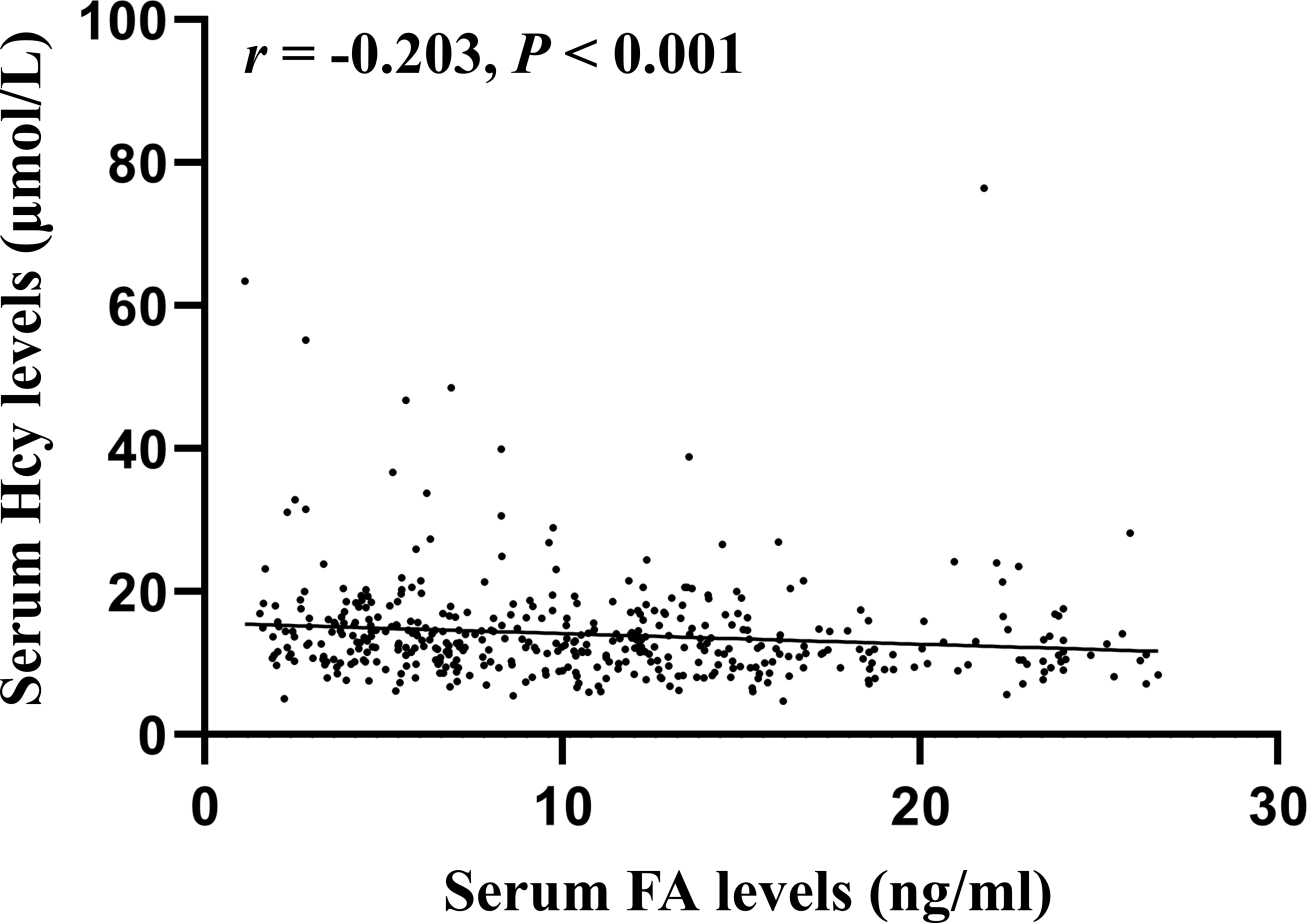
Correlation between serum Hcy and FA levels in Chinese urban population with hypertension.

Spearman correlation analysis showed significant correlations between Hcy levels and the following parameters: gender (*r* = -0.462, *P* < 0.001), MTHFR genotypes (*r* = 0.231, *P* < 0.001), DBP (*r* = 0.098, *P* = 0.036), heart rate (*r* = 0.093, *P* = 0.047), ALB (*r* = 0.136, *P* = 0.025), FA (*r* = -0.203, *P* < 0.001), Vitamin D (*r* = 0.131, *P* = 0.010), fruit consumption (*r* = -0.179, *P* < 0.001), type of meat (*r* = 0.100, *P* = 0.044), vitamin B12 supplements (*r* = -0.123, *P* = 0.012), smoking (*r* = 0.304, *P* < 0.001), frequency of alcohol consumed (*r* = 0.276, *P* < 0.001), and alcohol drinking status (*r* = 0.270, *P* < 0.001). **Figure 1** shows a scatter plot representing the correlation serum Hcy and FA levels. All variables except for DBP, ipulse, ALB, Vitamin D, type of meat, and vitamin B12 supplements passed Bonferroni correction (*P* < 0.05/30 = 0.0017). These significantly different variables from univariate analysis were included in multiple linear regression to detect correlates of Hcy levels (**Table 6**). Finally, the multiple linear regression revealed a significant association between Hcy levels and gender (*B* = -2.784, *t* = -2.931, *P* = 0.004), MTHFR genotypes (*B* = 1.410, *t* = 2.815, *P* = 0.005), and FA levels (*B* = -0.136, *t* = -2.192, *P* = 0.030).

**Table 6:** Associated factors for Hcy levels in Chinese urban population with hypertension based on multiple linear regression model.

Variables	Unstandardized B	Coefficient Std.error	Standardized coefficients Beta	*t*	*P*	VIF
Gender	-2.784	0.950	-0.239	-2.931	0.004	1.880
MTHFR genotypes	1.410	0.501	0.173	2.815	0.005	1.075
DBP	-0.067	0.035	-0.127	-1.903	0.059	1.257
Ipulse	0.053	0.029	0.124	1.863	0.064	1.251
ALB	0.023	0.052	0.027	0.433	0.658	1.023
FA	-0.136	0.062	-0.144	-2.192	0.030	1.231
Vitamin D	0.197	0.066	0.191	2.961	0.003	1.174
Fruit consumption	0.050	0.401	0.008	0.125	0.901	1.109
Type of meat	1.005	0.599	0.108	1.677	0.095	1.171
Vitamin B12 supplements	-1.411	0.976	-0.088	-1.445	0.150	1.050
Smoking	0.802	0.524	0.113	1.530	0.128	1.560
Frequency of alcohol consumed	1.583	0.939	0.193	1.686	0.094	3.729
Alcohol drinking status	-0.454	0.815	-0.065	-0.556	0.579	3.897

## Discussion

The objective of this study was to assess the prevalence, clinical correlation, and demographic characteristics of HHcy in the Chinese urban population with hypertension, with a focus on identifying risk factors for HHcy in hypertensive patients. The study yielded three key findings. Firstly, the prevalence of HHcy in the Chinese urban population with hypertension was determined to be 31.3%. Secondly, logistic regression analysis identified male gender and the presence of the MTHFR (TT) genotype as significant risk factors for HHcy. Lastly, the multiple linear regression revealed a significant association between Hcy levels and gender, MTHFR genotypes, and FA levels.

Multiple studies have examined the occurrence of HHcy in individuals with hypertension. One such study revealed that 73.3% of newly diagnosed hypertensive patients residing in seven urban communities in Nanjing, China, exhibited HHcy (19). Similarly, another study found a comparable prevalence of HHcy. Specifically, among 105 patients diagnosed with primary hypertension in Vietnam, 74.3% (78 patients) displayed elevated plasma total Hcy levels ≥ 15 µmol/L (20). The current investigation aimed to ascertain the prevalence of HHcy among the urban Chinese population with hypertension, revealing a rate of 31.3%. This finding closely aligns with the outcomes of a prior study conducted within China (13). Notably, a separate investigation involving 5935 hypertensive patients in China demonstrated a HHcy prevalence of 31.4% (13). Discrepancies in HHcy prevalence across studies may be attributed to various contextual factors, including population demographics, geographical location, and patient age. A meta-analysis conducted in China involving a sample size of 60,754 individuals aged 3-97 years revealed a positive correlation between age and the prevalence of HHcy, with the highest occurrence observed among individuals aged 65 years and above (21). Consequently, it is reasonable to hypothesize that the dissimilarity in HHcy prevalence between the present study and the study conducted in Nanjing (31.3% vs. 73.3%) could potentially be attributed to the variance in age distribution among the respective subject groups (54.1 vs. 63.3).

Previous evidence has indicated that gender plays a significant role in determining the levels of Hcy in humans. A study conducted on healthy individuals revealed that males tend to have higher serum Hcy levels compared to females (22). Moreover, a meta-analysis encompassing 36 studies and involving 60,754 subjects from 19 provinces and municipalities in China found that the prevalence of HHcy was notably higher in men than in women (21). Furthermore, elevated levels of serum homocysteine have been observed in male patients with hypertension (23), primary chronic venous disease (24), and psoriasis (25). Potential factors contributing to the observed sex-specific variation encompass disparities in muscle mass, estrogen levels, lifestyles, and vitamin levels. Specifically, Notably, the connection between Hcy production and creatine-creatinine synthesis is widely acknowledged. Given that males typically possess greater muscle mass, they exhibit an augmented requirement for creatine synthesis, consequently leading to increased Hcy production (26). Moreover, Chinese men exhibit a higher prevalence of alcohol consumption and cigarette smoking compared to women, both of which exhibit positive correlations with Hcy concentrations (27). Furthermore, the presence of hormonal disparities between males and females may also play a role in the observed sex-related discrepancy. Research has indicated that the administration of long-term hormone replacement therapy leads to decreased overall concentrations of Hcy in women who have undergone menopause (28). In the present study, the proportion of males was notably greater among individuals with HHcy compared to those without HHcy. Through the utilization of logistic regression analysis, male gender was identified as a significant risk factor for HHcy, while multiple linear regression analysis demonstrated a significant association between Hcy levels and gender. These findings offer additional empirical evidence establishing a correlation between gender and Hcy levels.

The enzyme MTHFR, which is dependent on folate, plays a significant role in the conversion of the amino acid homocysteine to methionine (29). The activity of MTHFR has a detrimental impact on the levels of Hcy in the serum (25). The MTHFR rs1801133 polymorphism involves a substitution of C to T at position 677, resulting in the conversion of alanine to valine. This missense mutation leads to a reduction of approximately 70% and 35% in the normal activity of the MTHFR enzyme in carriers with the TT and CT genotypes, respectively (30). Additionally, the TT allele is associated with increased levels of homocysteine in the serum (31). In this study, CT and TT genotypes of MTHFR C677T polymorphism increased the risk of HHcy compared to CC genotype. Additionally, logistic regression analysis revealed that the presence of the MTHFR (TT) genotype was a significant risk factor for HHcy. Furthermore, multiple linear regression analysis demonstrated a significant association between homocysteine levels and the MTHFR genotype. Given that approximately 25% of the Chinese population possesses the MTHFR C677T TT genotype (32), the assessment of MTHFR genotype within the Chinese populace, particularly among individuals with hypertension, holds significant clinical implications in terms of cardio-cerebrovascular disease prevention and treatment.

Presently, the utilization of FA and B vitamins is employed to attain desired Hcy levels, exhibiting evident efficacy in diminishing plasma Hcy concentrations (33). While not yet substantiated, the reduction of overall plasma Hcy levels via FA and/or vitamin B12 intake may potentially mitigate the susceptibility to vascular diseases among HHcy individuals (34). Another clinical trial investigation has documented that the addition of FA as a supplementary therapy, administered at a dosage of 5 mg per day, exhibits the potential to markedly decrease serum Hcy levels (35). This intervention may play a contributory role in the pathogenesis of vascular disorders, including Buerger’s disease (35). In the current investigation, individuals diagnosed with HHcy exhibited notably diminished levels of FA in comparison to those without HHcy. Regarding lifestyle habits, individuals with HHcy demonstrated a reduced intake of vitamin B12 in comparison to those without HHcy. Furthermore, inverse associations were observed between Hcy levels and both FA levels and vitamin B12 supplementation. Additionally, fruits and vegetables are recognized as abundant sources of FA and B vitamins, which contribute to elevated plasma levels of these vitamins and decreased Hcy levels (36). In the present study, it was consistently observed that individuals with HHcy exhibited lower levels of fruit intake compared to those without HHcy. These findings imply that augmenting FA levels and incorporating vitamin B12 supplements could potentially decrease Hcy levels in hypertensive patients, consequently reducing the incidence of cardio-cerebrovascular disease.

Evidence consistently supports the notion that smoking, alcohol consumption, and physical inactivity have a significant impact on elevating Hcy levels (37, 38). Consequently, these lifestyle factors were incorporated into the current study to ascertain the specific risk factors associated with HHcy in hypertensive individuals. Correspondingly, our findings revealed a heightened prevalence of tobacco smoking and alcohol use among subjects with HHcy, thereby reinforcing the recommendation for hypertensive patients with HHcy to adopt lifestyle modifications such as reducing alcohol intake and quitting smoking.

Several limitations to this study need to be acknowledged. First, this is a cross-sectional study design and cannot show a causal relationship between the presence of HHcy and a given risk factor. Second, this study is limited by its observational design and small subset size, which reduces the power of the statistical analysis. Third, generalizability was limited due to the fact that the study was performed in only one region of China. Fourth, an additional limitation of this study pertains to the absence of validation for the questionnaire tool and the omission of a reliability assessment. Fifth, given that diuretics decrease the absorption of FA, the absence of precise categorizations pertaining to the types of antihypertensive drugs is regarded as a limitation of this research.

In conclusion, our study reveals a notable occurrence of HHcy among the Chinese urban population with hypertension, those at greatest risk are male, genotype MTHFR 677TT, and a decreased FA level. Consequently, it is imperative to implement future strategies for managing and intervening in these factors to mitigate the development of H type hypertension and minimize associated cardio-cerebrovascular events within this population. For example, to mitigate the occurrence of HHcy and cardio-cerebrovascular disease, particularly among male individuals with the MTHFR C677T TT genotype, it is recommended to implement pharmacotherapy and/or lifestyle adjustments encompassing physical activity, nutrition, and dietary supplementation, such as FA. Notably, HHcy has been considered a specific disease in the Chinese Han population (39). The population with the TT genotype accounts for 25% of the Chinese Han population, whereas the population with the TT genotype in North America and Europe accounts for only approximately 12% of the combined population (40). Thus, the risk factors for HHcy identified in the Chinese population in this study, specifically the MTHFR677TT genotype, may not be applicable to other populations, such as North Americans and Europeans.

## Data availability statement

The original contributions presented in the study are included in the article/supplementary material. Further inquiries can be directed to the corresponding author.

## Ethics statement

The studies involving humans were approved by Clinical Research Ethics Committee of Shenzhen Second People’s Hospital. The studies were conducted in accordance with the local legislation and institutional requirements. The participants provided their written informed consent to participate in this study.

## Author contributions

YX: Conceptualization, Data curation, Formal analysis, Investigation, Methodology, Project administration, Software, Validation, Writing – original draft. HF: Investigation, Methodology, Validation, Writing – original draft. LZ: Software, Supervision, Writing – original draft. YL: Formal analysis, Resources, Software, Writing – original draft. FC: Conceptualization, Data curation, Funding acquisition, Project administration, Resources, Supervision, Writing – review & editing. LR: Conceptualization, Funding acquisition, Resources, Supervision, Validation, Visualization, Writing – review & editing.
